# Ratcheting Strain and Microstructure Evolution of AZ31B Magnesium Alloy under a Tensile-Tensile Cyclic Loading

**DOI:** 10.3390/ma11040513

**Published:** 2018-03-28

**Authors:** Zhifeng Yan, Denghui Wang, Wenxian Wang, Jun Zhou, Xiuli He, Peng Dong, Hongxia Zhang, Liyong Sun

**Affiliations:** 1 Shanxi Key Laboratory of Advanced Magnesium-Based Materials, School of Materials Science and Engineering, Taiyuan University of Technology, Taiyuan 030024, China; yanzhifeng@tyut.edu.cn (Z.Y.); 18503437855@163.com (D.W.); wwx960@126.com (W.W.); juz17@psu.edu (J.Z.); 2 School of Engineering, The Behrend College, Pennsylvania State University Erie, Erie, PA 16563, USA; lus28@psu.edu; 3 Department of Mechanical Engineering, Taiyuan Institute of Technology, No. 31, Xinlan Road, Taiyuan 030008, China

**Keywords:** AZ31B magnesium alloy, ratcheting strain, microstructure change

## Abstract

In this paper, studies were conducted to investigate the deformation behavior and microstructure change in a hot-rolled AZ31B magnesium alloy during a tensile-tensile cyclic loading. The relationship between ratcheting effect and microstructure change was discussed. The ratcheting effect in the material during current tensile-tensile fatigue loading exceeds the material’s fatigue limit and the development of ratcheting strain in the material experienced three stages: initial sharp increase stage (Stage I); steady stage (Stage II); and final abrupt increase stage (Stage III). Microstructure changes in Stage I and Stage II are mainly caused by activation of basal slip system. The Extra Geometrically Necessary Dislocations (GNDs) were also calculated to discuss the relationship between the dislocation caused by the basal slip system and the ratcheting strain during the cyclic loading. In Stage III, both the basal slip and the {11−20} twins are found active during the crack propagation. The fatigue crack initiation in the AZ31B magnesium alloy is found due to the basal slip and the {11−20} tensile twins.

## 1. Introduction

Magnesium alloys have great potential in many engineering applications because of the great characteristics, such as low density and easily recycled performance, etc. [1,2]. In addition, many components used in engineering are usually subjected to alternating loads. Therefore, it is significant to master the fatigue properties of magnesium alloys [3,4,5]. 

The alloys deform inelastically or display certain ratcheting effect under asymmetric alternating loads, which could lead to further large deformation and damage [6]. For stress-controlled asymmetric cyclic deformation experiments, the ratcheting behavior of Mg alloys depends greatly upon applied mean stress [7,8,9]. The ratcheting strain and its accumulation rate in the material increase as the stress increases, and then the damage accelerates and the fatigue life reduces [10]. The uniaxial stress-controlled fatigue behavior of the AZ91 alloy has been studied [11]. Cyclic deformation of an alloy occurs along with micro deformation [12]. Deformation mechanisms caused by the dislocation slip and twinning under a low-cycle loading of the magnesium alloys have also been studied [13]. Dynamic recrystallization in the Mg–6Zn–1 Mn alloy was also found under a fatigue loading [14]. To achieve an arbitrary homogeneous deformation in a polycrystalline material, five independent slip systems are required to be activated [15,16]. A wide range of crystal orientations was observed in the post-fatigued specimens, leading to a significant weakening of the basal texture [17]. Specimen microstructure changes because of the activated slip and twin system under alternating loads [18,19,20]. At room temperature, it is difficult to activate the non-basal slip system due to a large critical resolved shear stress (CRSS) [21]. 

During a servo-hydraulic fatigue test, since the alloy is under complex stress, it takes long time to reach the material’s fatigue limit (at 10^7^ cycles). In recent years, microstructure changes were reported only after fatigue test, however its evolution during the fatigue test was not found. In the present paper, cyclic deformation behaviors of (hot-rolled) AZ31B alloy under tensile-tensile cyclic loads are investigated at ambient temperature. The relationship between the ratcheting effect and microstructure change under a cyclic loading are discussed.

## 2. Experiments

**Figure 1:**
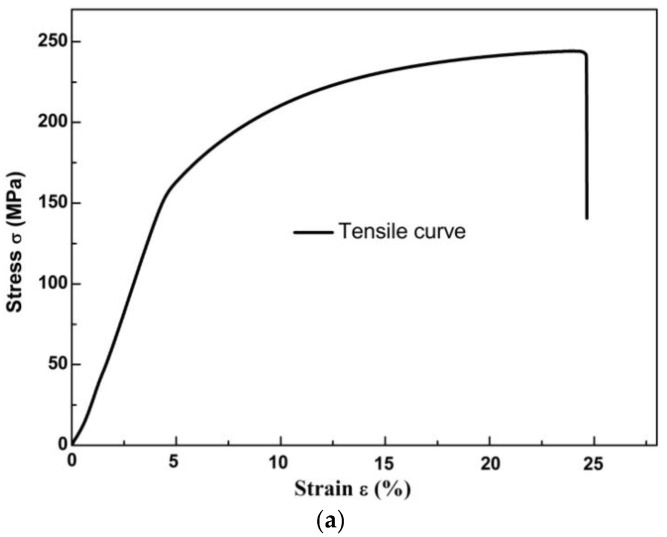

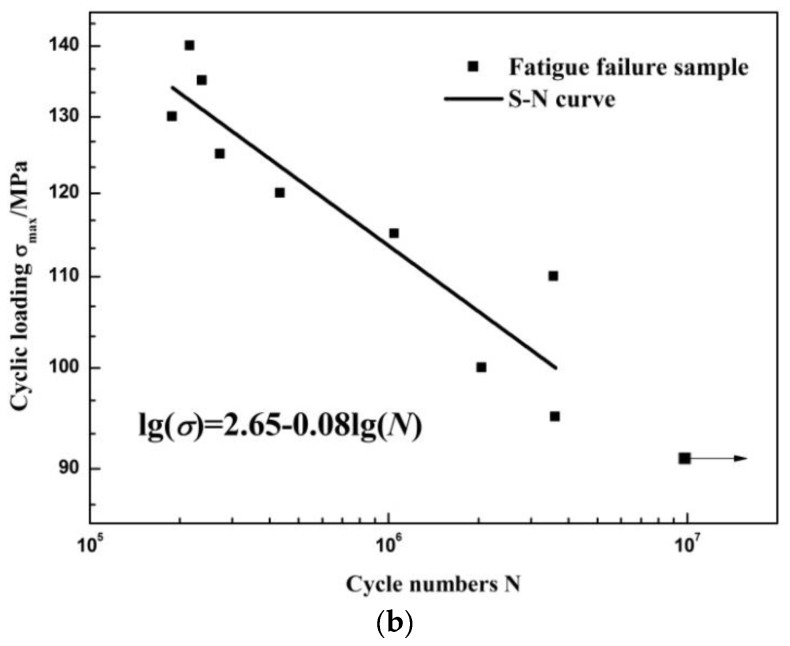
The strength curves of AZ31B magnesium alloy: (**a**) The tensile stress–strain curve; (**b**) *S*–*N* curve.

In the experiment, the AZ31B (hot-rolled) alloy plate, 5 mm thick, was used. Its chemical compositions (in wt. %) are 2.83Al, 0.74Zn, 0.06Mn and balance Mg. All experiments were conducted at room temperature. Both tensile and fatigue specimens were first cut from the AZ31B magnesium alloy plate along its extrusion direction, and then polished with metallographic sandpapers of different grades (800, 1000 and 1500 mesh successively). The curve between the stress and strain the alloy under tensile loading is shown in Figure 1a. It can be seen that the tensile strength of the alloy is 240.12 MPa, the yield strength with average 0.2% offset is 146.02 MPa. The cyclic strength at 10^7^ cycles is 95.05 MPa, as shown in Figure 1b. The gage diameter of the fatigue specimens cut from the plate is 25 mm (Chinese Standard GBT 3075-2008 [22]). The specimens’ surfaces were polished using emery papers, which was to reduce the external effects.

The fatigue tests were carried out on an electro-hydraulic servo-controlled machine (SDS100), the sinusoidal-waveform is adopted and it is stress-controlled, the stress ratio (*σ_min_*/*σ_max_*) is 0.1 and the frequency is 1 Hz. The specimen tested at *σ_max_* 140 MPa was investigated representatively, and its deformation and microstructure were analyzed during the whole fatigue process.

Electron backscattered diffraction (EBSD, JEOL JSM-7800F) was conducted to analyze and compare the microstructures of the base metal and specimen after fatigue test, and their cycles were different. The EBSD samples were taken in the surface of the gage regions of the specimens after fatigue testing. The samples were processed with SiC paper (1200 grit), and then they were etched using acetic picral. The data was collected and analyzed using the system’s data acquisition and analysis software (HKL Channel 5, Oxford Instruments, Abingdon, UK).

## 3. Results and Discussion

### 3.1. Deformation Curves of the AZ31B Alloy under cyclic loading

**Figure 2:**
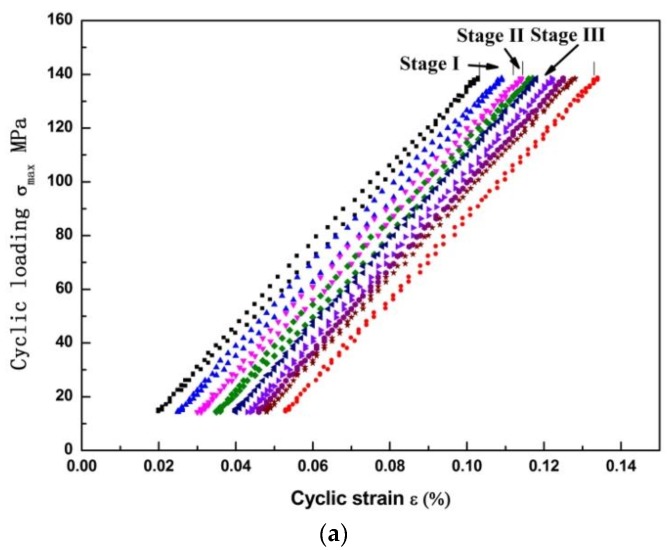

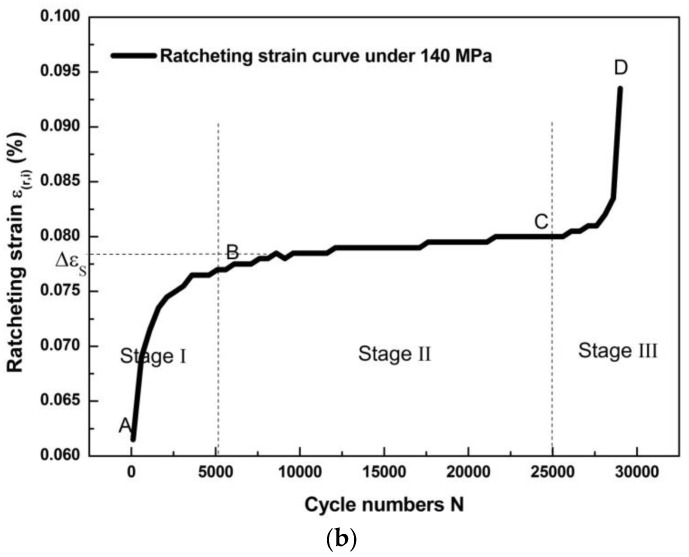
The deformation curves under *σ*_max_ of 140 MPa in AZ31B magnesium specimen: (**a**) The hysteresis loops under different cycles; (**b**) The ratcheting evolution curve under different cycles.

The hysteresis loops of the AZ31B magnesium alloy under the cyclic load of 140 MPa are shown in Figure 2. As shown in the results, though the load is below the yield strength of the AZ31B magnesium alloy (*σ*_0.2_=146.02 MPa), the irreversible deformation still occurred. During the initial loading process, the hysteresis loops’ change was unstable. The irreversible deformation increased with the cycles in the material. After 10,000 cycles, the hysteresis loops remained narrower and stable. This means that the clear ratcheting effect occurred during the cyclic loading. The ratcheting strain is defined as the strain $\varepsilon_{\left(r,i\right)}$ at cycle “*i”* [23]:

(1) $$\varepsilon_{\left(r,i\right)}=\frac{\varepsilon_{\left(i,\max\right)}+\varepsilon_{\left(i,\min\right)}}{2},$$

where *ε*_(*i*,max)_ is the maximum strain and *ε*_(*i*,min)_ is the minimum strain at cycle “*i”*. 

The relationship between the ratcheting curve and cycle number is given in Figure 2b. The ratcheting strain experienced three stages: (1) the sharp increase stage (Stage I) which occurred in less than cycles of 5000; (2) steady stage (Stage II) with cycle numbers between 5000 and 25,000; and abrupt increase stage (Stage III) with cycle numbers over 25,000. The ratcheting strain in the first stage is increase sharply. The strain Δ*ε*_s_ keep constant in the second stage. In Stage III, the ratcheting strain speed was accelerated by the crack open distance during the crack propagated in the material, causing the final fracture [24]. As noticed, most of the fatigue life was spend before the Stage III. Especially, the strain was almost unchanged in Stage II. Furthermore, the ratcheting curves have the same tendency under different stress levels [25]. Therefore, the relationship between the ratcheting strain and microstructure evolution is the main concern of current study.

### 3.2. Microstructure Evolution

The ratcheting strain development in magnesium alloy is associated with microstructure change, such as the interactions between the dislocation movement and twinning [26]. 

**Figure 3:**
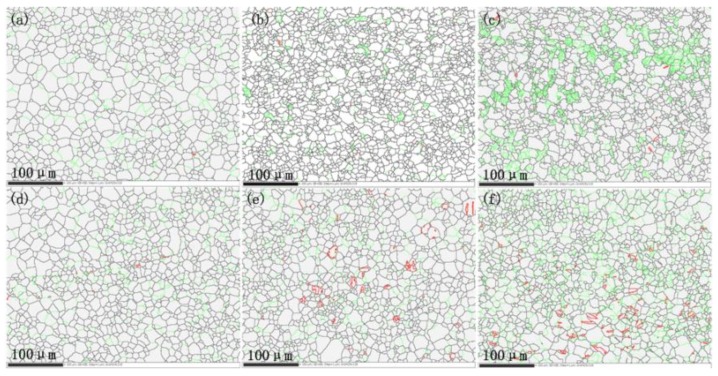
The grains change during cyclic loading: (**a**) As-received material; (**b**) cycles at 1000; (**c**) cycles at 5000; (**d**) cycles at 10,000; (**e**) cycles at 20,000; (**f**) cycles at 30,000.

The microstructure change during the cyclic loading was analyzed according to the EBSD, and the results are shown in Figure 3. The microstructures shown are for the material in the gauge regions (away from the cracks). In Figure 3, the green lines are the misorientation angle around 2°, and the red lines are the misorientation angle around 86.3°. The evolution of misorientation angle will be discussed later.

As shown in Figure 3a, the equiaxed grains range from 1 to 32 μm is contained in the as-received material, which the grains is inhomogeneous. The grain size quickly decreased around 5000 cycles. The dynamic recrystallization is believed to cause the grain refinement during the fatigue process [21]. The specimen did not undergo macroscopic plastic deformation during the stress-controlled cyclic loading, and the surface temperature of the specimen was below 50 °C [27]. Therefore, the DRX mechanism in this study is different from the conventional hot deformation such as rolling and extruding process [28]. The fatigue behavior of magnesium alloy can be understood according to the Continuous dynamic recrystallization (CDRX). 

The CDRX is the variation of low-angle grain boundaries (LAGBs) which the angle θ ≤ 15°. It involves the LAGBs formation of and the transformation to the high-angle grain boundaries (HAGBs). And the relationship between the change of the grain boundaries and the decreased of the grain size [15].

**Figure 4:**
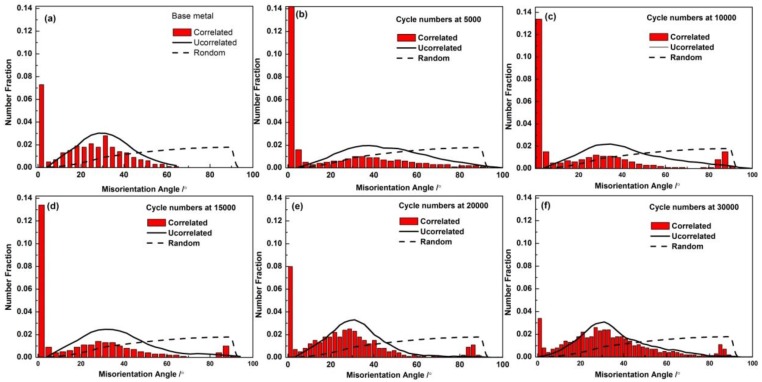
Misorientation distributions in AZ31B magnesium alloy: (**a**) as-received metal; (**b**) cycles at 5000; (**c**) cycles at 10,000; (**d**) cycles at 15,000; (**e**) cycles at 20,000; (**f**) cycles at 30,000.

The grain boundary misorientation angle distributions in AZ31B magnesium alloys during cyclic loading are shown in Figure 4, respectively. In these figures, the ‘‘Correlated’’ means misorientations tested according to the neighboring points, and the ‘‘Uncorrelated’’ shows the calculated misorientations by using the random points during the scan, ‘‘Random’’ implies the theoretically misorientation distribution of a random texture [29]. 

It is noted that, in Figure 4a, a preferred orientation or a strong texture in the magnesium alloy is formed, according to the gaps between the uncorrected and the random curves in abscissa around 30°. The fraction of this preferred orientation remained high during the fatigue process. 

The aforementioned grain boundary change was related to the movement of the slip system. In Figure 4a, the “correlated” misorientation angle around 2° is noticed taking the largest fraction in AZ31B magnesium alloy. During the Stage I, as shown in Figure 4b, the fraction increased sharply. However, with the increase of cycles (from Stage II to Stage III), the percentage of the LAGBs decreased and LAGBS changed to HAGBs gradually, as shown in Figure 4c–f. 

The fraction of the misorientation angle around 30° is also found relatively high. This should be caused by the active slip system at 30° <0001>, since for magnesium with a 6-fold crystallographic symmetry of hexagonal close-packed structure, the most difference of orientation between the two ideal (0001) grains is 30° [30]. In Stage II, a few 86° twinning occurred as shown in Figure 4c. Since only {10−12} twining could lead to a 86.3° reorientation of the crystal lattice, this clearly indicates that the main activated twin is {10−12} tensile twinning. During the fatigue loading, the grain boundary misorientation became more and more uniform. Similar results were also reported in other studies [31]. According to these texture changes shown, it can be concluded that the active basal slip system is the main deformation mechanism before Stage III.

**Figure 5:**
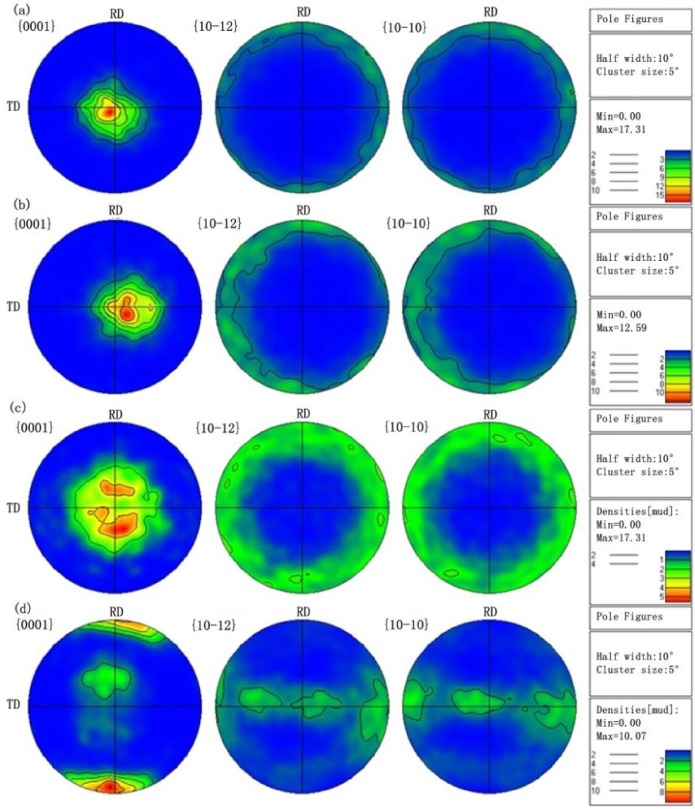
The pole figures of AZ31B magnesium alloy under cycles: (**a**) Base material; (**b**) Point ‘B’ in Figure 2b; (**c**) Point ‘C’ in Figure 2b; (**d**) Point ‘D’ in Figure 2b.

The evolution of the texture under different cycles according to the pole figures are given in Figure 5. As shown in Figure 5a, the as-received magnesium alloy has a typical {0001} texture with a high density of 17.31. As shown in Figure 5b to Figure 5c, the texture is almost the same in Stage I and Stage II. But a much wider spread of orientations was noticed in the post-fatigued specimens. The texture intensity dropped from 17.31 to 5.62, which means a significant weakening of the basal texture from Stage I to Stage II. In Stage III, the texture type changed from basal texture to prismatic texture with the density of 10.7, shown in Figure 5d. In general, the texture was weakened with the increasing cycles.

### 3.3. Geometrically Necessary Dislocation (GND) Density Change with the Cycle Increase

**Figure 6:**
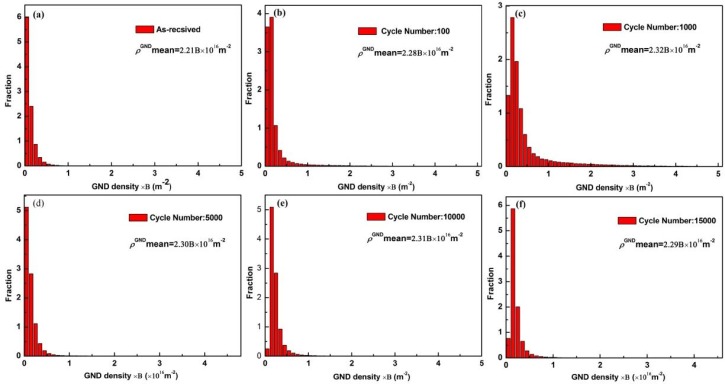
The mean GND density distributions under loading of 140 MPa (**a**) as-received magnesium alloy; and in specimens (**b**) cycles at 100; (**c**) cycles at 1000; (**d**) cycles at 5000; (**e**) cycles at 10,000; (**f**) cycles at 15,000.

In order to analyze the dislocation density change with the cycle increase, the kernel average misorientation (KAM) was calculated according to the local misorientation tests data [32]. The GND evolution was studied by measuring the local crystal orientations in the surface of the material [33]. Local misorientation angles greater than 2° are excluded in the present paper. According to its 24 surrounding points, the local misorientation was determined as follows:

(2) $$\Delta \theta_{i}=\frac{1}{n}\sum_{j=1}^{n}\left|\theta_{j}^{sur}-\theta_{i}\right|,$$

where *θ*_i_ is the local misorientation around the point ‘*i*’ (100 nm × 100 nm) and $\theta_{j}^{sur}$ is the misorientation at its neighboring point ‘*j*’. To calculate the GND density, a simple equation from the strain gradient theory is used [34,35]:

(3) $$\rho^{\mathrm{GND}}=\frac{2\Delta \theta_{i}}{ub}=B\Delta \theta_{i}$$

where *ρ*^GND^ represents the GND density in tested area; Δ*θ_i_* means the local misorientation; *u* is the unit length of the point (100 nm); *b* is the Burgers vector. $B=\frac{2}{ub}=2b\times 10^{16}m^{2}$, is a constant for specific material. The column charts of the calculated GND density and their change were given in Figure 6.

**Figure 7:**
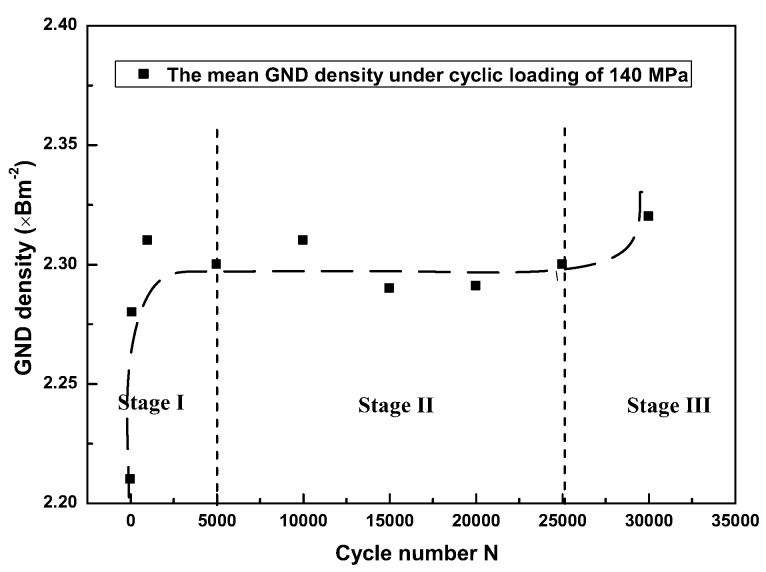
The mean GND density distributions under 140 MPa.

The mean GND density distributions with cycles are shown in Figure 7. The dotted line in the figure is a fitting curve according to the density of GNDs under different cycles and *l*_1_ and *l*_2_ are the dividing lines of the three stages. The GND density increased sharply in the first Stage and fluctuated in small range in Stage II. The evolution pattern of the GND density is consistent with that shown in the ratcheting curve in Figure 2b. That is, the deformation of the material under cyclic loading was strongly affected by the change of the dislocation density.

### 3.4. The Microstructure at the Crack Tip Area

**Figure 8:**
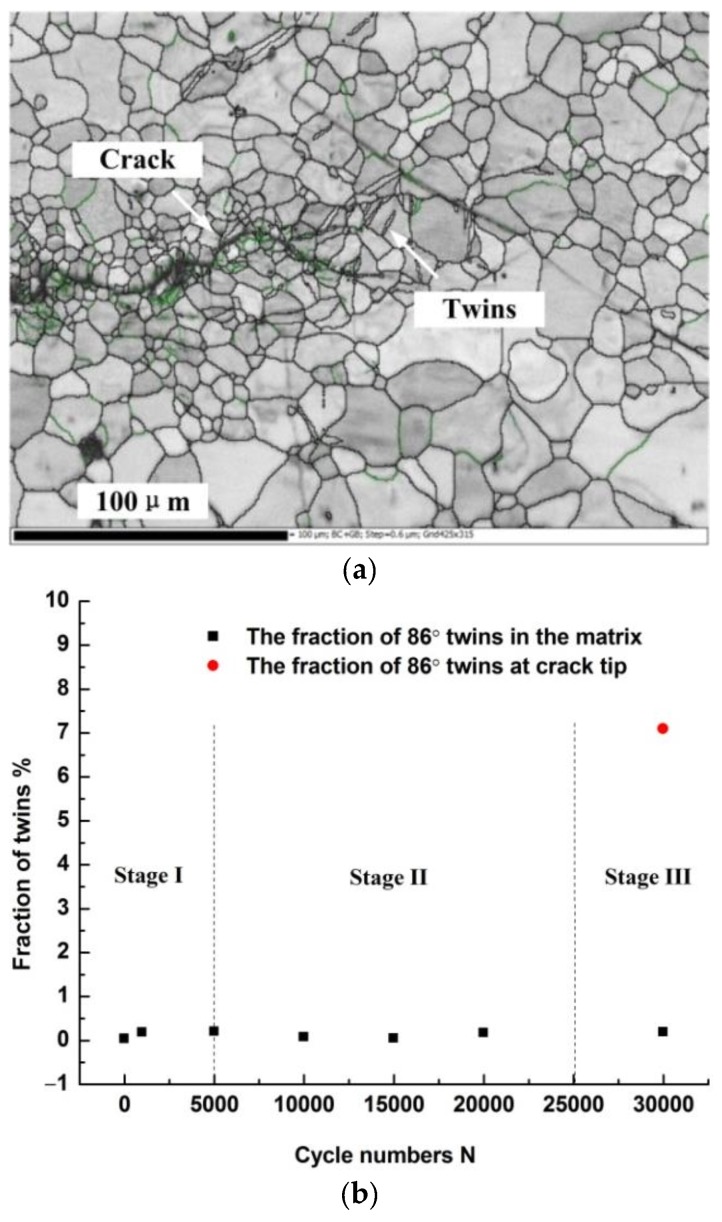
(**a**) The microstructure at the crack tip; (**b**) The content of 86° twins.

The main deformation during the crack propagation was concentrated at the crack tip (in Stage III). Based on fracture mechanics analysis, the stress at the crack tip is infinite theoretically (singularity appears), but the actual material will always yield at a certain stress and will produce plastic deformation zone around the crack tip. The microstructure at the crack tip can be seen in Figure 8a. The grain is refined at the edge of the crack. Both LAGBs and HAGBs indicating preferred orientation or formation at the crack tip. The content of {11−20} twins in the matrix is relatively low, but the content of these twins at the crack tip increases sharply, as shown in Figure 8b. 

### 3.5. The Fatigue Fracture Mechanism

As discussed above, the fatigue fracture of AZ31B magnesium alloy is associated with the corresponding change of slip systems. The polycrystalline material, which need at least 5 <a> slip systems independently, could deformed homogeneously. They distributed on the two (0001) basal plane and three non-basal plane slip systems, {10−10} prismatic plane and {10−11} pyramidal plane together with a <c+a> slip on {10−12} pyramid al plane, respectively. Thus the slip systems in Mg could fulfill such a criterion theoretically [19]. For the low critical resolved shear stress (CRSS) at room temperature, the basal slip system is easy to be activated [21]. Therefore, deformations in Stage I and Stage II are mainly caused by the {0001} basal slip.

**Figure 9:**
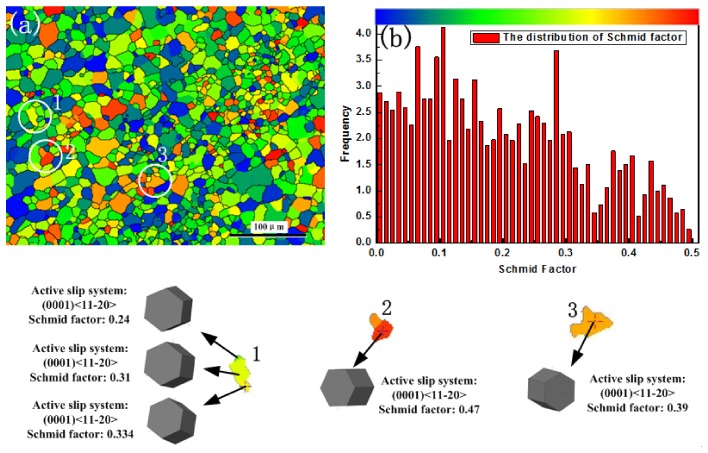
The Schmid factor distribution in the as-received material. (**a**) the distributions of the Schmid factor on the base metal; (**b**) the crystal shifts from hard orientation to soft orientation

However, micro-deformation ability is non-uniform in a multi-crystal material. Figure 9a shows the distributions of the Schmid factor on the base metal. The various crystal color represents various deformation ability of the crystal. As can been seen from the histogram distribution of the Schmid factor, when the Schmid factor increases in the range of 0–0.5, the crystal shifts from hard orientation to soft orientation shown in Figure 9b. It can also be found that the distributions of the Schmid factor are unevenly for the base metal, and a majority of the crystals distributed in hard orientations uniformly (The max distribution frequency around 0.1). Furthermore, the Schmid factors are noticed diverse in different crystals, as shown in the enlarged crystals 1, 2 and 3, respectively. The crystals having different Schmid factors can cause unmatched deformation ability in the alloy under an alternating load. The activation of basal slip depends on the stress amplitude. Therefore, the GND density remains constant in Stage II.

In Stage III during the crack propagation, large deformation is caused by the non-basal slip. For magnesium alloys the tensile twins, prismatic slips, and <c+a> pyramidal slips are the main deformation modes. Among them, the critical resolved shear stress (CRSS) of the tensile twin is much lower than that of prismatic and pyramidal slips, as shown in Figure 8. According to above discussions, fracture of the AZ31B alloy sheet under a tensile-tensile cyclic loading is determined by the basal plane slip and the tensile twins in the alloy.

## 4. Conclusions

The tensile-tensile cyclic loading test of the AZ31B alloy was conducted, the deformation and microstructure were analyzed. Conclusions are listed below:

(1) There is the ratcheting effect for the alloy under a cyclic loading of 140 MPa. The development of the ratcheting strain includes three stages: rapid increase stage (Stage I); steady stage (Stage II); and final abrupt increase stage (Stage III). The change of Extra Geometrically Necessary Dislocations (GNDs) was found to have the same evolutionary tendency as that of the ratcheting strain.
(2) Two deformation mechanisms, basal slip and {10−12} tensile twinning, are found to be dominant during a cyclic loading, which is stress-controlled. The {0001} basal slip dominates plastic deformation during the crack initiation (Stage I and II) and the 86° {10−12} tensile twinning is dominant in Stage III.
